# Elevating Phospholipids Production *Yarrowia lipolytica* from Crude Glycerol

**DOI:** 10.3390/ijms231810737

**Published:** 2022-09-14

**Authors:** Patrycja Szczepańska, Magdalena Rychlicka, Paweł Moroz, Tomasz Janek, Anna Gliszczyńska, Zbigniew Lazar

**Affiliations:** 1 Department of Biotechnology and Food Microbiology, Wroclaw University of Environmental and Life Sciences, Chelmonskiego 37, 51-630 Wroclaw, Poland; 2 Department of Food Chemistry and Biocatalysis, Wroclaw University of Environmental and Life Sciences, Chelmonskiego 37, 51-630 Wroclaw, Poland

**Keywords:** phospholipids, oleaginous yeast, *Yarrowia lipolytica*, neutral lipids, membrane lipids, glycerol, crude glycerol, metabolic engineering

## Abstract

Phospholipids (PLs) are a class of lipids with many proven biological functions. They are commonly used in lipid replacement therapy to enrich cell membranes damaged in chronic neurodegenerative diseases, cancer, or aging processes. Due to their amphipathic nature, PLs have been widely used in food, cosmetic, and pharmaceutical products as natural emulsifiers and components of liposomes. In *Yarrowia lipolytica*, PLs are synthesized through a similar pathway like in higher eukaryotes. However, PL biosynthesis in this yeast is still poorly understood. The key intermediate in this pathway is phosphatidic acid, which in *Y. lipolytica* is mostly directed to the production of triacylglycerols and, in a lower amount, to PL. This study aimed to deliver a strain with improved PL production, with a particular emphasis on increased biosynthesis of phosphatidylcholine (PC). Several genetic modifications were performed: overexpression of genes from PL biosynthesis pathways as well as the deletion of genes responsible for PL degradation. The best performing strain (overexpressing CDP-diacylglycerol synthase (*CDS*) and phospholipid methyltransferase (*OPI3*)) reached 360% of PL improvement compared to the wild-type strain in glucose-based medium. With the substitution of glucose by glycerol, a preferred carbon source by *Y. lipolytica*, an almost 280% improvement of PL was obtained by transformant overexpressing *CDS*, *OPI3*, diacylglycerol kinase (*DGK1*), and glycerol kinase (*GUT1*) in comparison to the wild-type strain. To further increase the amount of PL, the optimization of culture conditions, followed by the upscaling to a 2 L bioreactor, were performed. Crude glycerol, being a cheap and renewable substrate, was used to reduce the costs of PL production. In this process 653.7 mg/L of PL, including 352.6 mg/L of PC, was obtained. This study proved that *Y. lipolytica* is an excellent potential producer of phospholipids, especially from waste substrates.

## 1. Introduction

Phospholipids (PLs) are the main structural components of cell membranes and are essential for vital cellular processes, such as vesicle formation, membrane fusion, and cell division [1]. PLs belong to the class of complex lipids with amphiphilic nature. Their structure is composed of the glycerine-3-phosphate backbone in which hydrophobic fatty acyl groups are esterified to the sn-1 and sn-2 positions [2]. The sn-3 position consists of a phosphate group that contributes hydrophilicity. The simplest PL is phosphatidic acid (PA) and all others are named after the hydrophilic residue attached to the phosphate group. Four main groups of PL have been identified: ethanolamine, inositol, serine, and choline. These groups form the most biologically important phospholipids, which are phosphatidylethanolamine (PE), phosphatidylinositol (PI), phosphatidylserine (PS), and phosphatidylcholine (PC) [3].

PLs show many health benefits; e.g., they regulate the function of many organs, decrease the level of cholesterol and triglycerides in blood, repair damaged liver tissue, and prevent neurological diseases [4]. Moreover, PLs are commonly used in lipid replacement therapy to enrich cell membranes that are damaged in chronic neurodegenerative diseases, cancer, or aging processes. Due to their amphipathic nature, PLs have been widely used in food, cosmetic, and pharmaceutical products as natural emulsifiers and components of liposomes [5].

With the advent and rapid development of metabolic engineering and synthetic biology, the number of microbe-based cellular factories is growing rapidly, promising the ability to convert renewable carbon sources into a wide range of chemicals, biofuels, pharmaceuticals, and natural bioactive compounds [6]. Oleaginous yeasts are characterized by complex inner membranes that allow the storage of large amounts of neutral lipids (mainly triacylglycerols-TAGs). These yeasts grow rapidly and produce lipids at high rates [7], thus becoming a viable platform for the production of valuable lipid compounds. The model oleaginous yeast *Yarrowia lipolytica* has been reported to enhance lipid production [8,9]. Engineering strategies undertaken to date have mainly focused on the biosynthesis of TAGs or free fatty acids. However, there have been no efforts to overproduce PL by engineering their metabolism, which is quite poorly understood and characterized by structural diversity, tight genetic regulation, or involvement of multiple organelles. The well-characterized *Y. lipolytica*, having a GRAS (generally recognized as safe) status, being amenable to genetic engineering due to the availability of various genome editing tools, and being resistant and tolerant to harsh fermentation conditions, may become a promising platform for the synthesis of PL. Moreover, *Y. lipolytica* has the ability to utilize crude glycerol, which contains many impurities including heavy metals, methanol, and salts [10], resulting in a low market price for this carbon source.

This study aimed to decipher PL metabolism in *Y. lipolytica* and increase their production through a series of genetic engineering manipulations. Special attention was put on the improvement of PC biosynthesis. First, strains of *Y. lipolytica* characterized by the increased production of PL were constructed, which were then used in research related to the optimization of the production of these compounds. The influence of the carbon source (glucose, glycerol), nitrogen availability, cultivation time, and the dependence of enzymes in the PL biosynthesis pathway on the availability of NADPH were analyzed. Finally, bioreactor scale cultures were carried out using raw glycerol as a carbon source.

## 2. Results and Discussion

### 2.1. Establishing the Y. lipolytica as a Phospholipid-Producing Platform Relative to Different Carbon Sources

**Figure 1:**
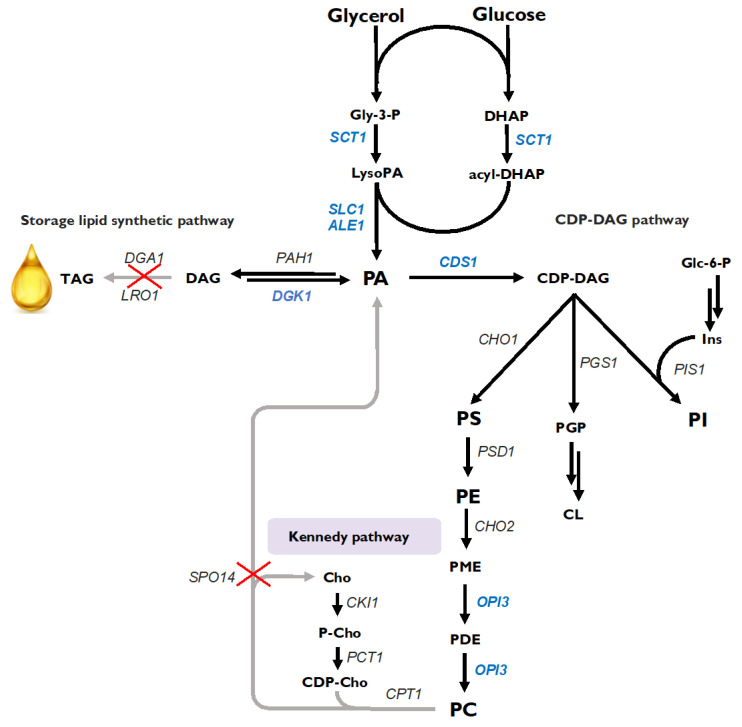
Engineering a phospholipid platform in *Yarrowia lipolytica*. Marked in blue, overexpressed genes; grey arrows marked with X, deleted genes. DHAP, dihydroxyacetone phosphate; Gly-3-P, glycerol 3-phosphate; DAG, diacylglycerol; TAG, triacylglycerol; PME, phosphatidylmonomethylethanolamine; PDE, phosphatidyldimethylethanolamine; PGP, phosphatidylglycerophosphate; CL, cardiolipin; Cho, choline; Ins, inositol; PME, phosphatidylmonomethylethanolamine.

Several genetic modifications were performed to construct strains for enhanced phospholipid production, mainly for phosphatidylcholine: the overexpression of genes involved in the PL biosynthesis pathway (shown in blue) and deletion of genes responsible for PL degradation (shown by red crosses; Figure 1). *De novo* lipid synthesis begins with the acylation of Gly-3-P by glycerol-3-phosphate acyltransferase encoded by *SCT1* (YALI0C00209g), resulting in the formation of lysophosphatidic acid (LysoPA), which is acylated to phosphatidic acid (PA) by lysophospholipid acyltransferases encoded by *SLC1* (YALI0E18964g) and *ALE1* (YALI0F19514g). A key intermediate in phospholipid biosynthesis is PA, which in *Y. lipolytica* is mainly directed to TAG production and, in smaller amounts, to the cytidine diphosphate diacylglycerol (CDP-DAG) pathway, in which membrane phospholipids are synthesized [Figure 1]. 

**Figure 2:**
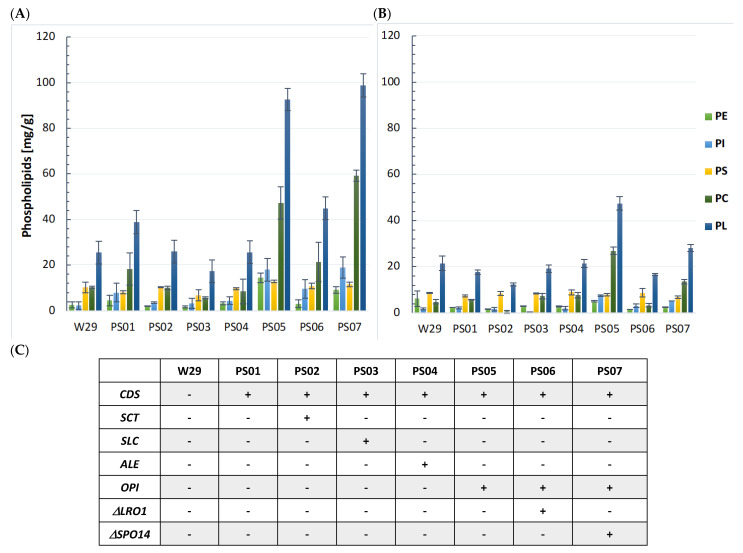
Production of phospholipids by engineered *Y. lipolytica* strains. Error bars represent standard deviation from at least three biological replicates. (**A**) Phospholipid levels of engineered strains in shake flasks with YNB minimal medium and 60 g/L glucose, C/N 99. Titers obtained after 144 h cultivation at 160 rpm and 28 °C. (**B**) Phospholipid levels of engineered strains in shake flasks with YNB minimal medium and 60 g/L glycerol, conditions as when cultured on glucose. (**C**) Summary table of genes overexpressed/deleted in each of the evaluated strains.

Therefore, we first constructed strains in which the overall amount of PA would be increased. It was achieved through the overexpression of the individual genes *SCT1*, *SLC1*, and *ALE1,* and redirecting a larger pool of PA toward the CDP-DAG pathway by overexpressing CDP-DAG synthase (*CDS*), which encodes the conversion of PA to liponucleotide CDP-DAG. However, the phospholipid profile of strains PS01, PS02, PS03, and PS04, in both glucose and glycerol based cultures, was the same or slightly changed (PL lowered) compared to the wild-type strain W29 [Figure 2A,B]. This may suggest that PA levels are tightly regulated in the cell by phosphatidic acid phosphatase *PAH1* (YALI0D27016g), which catalyzes the production of diacylglycerol (DAG) from PA in a reaction that is thought to be a major step in TAG biosynthesis [11]. During increased PA production, the Pah1 enzyme becomes more active and efficiently dephosphorylates larger amounts of substrate into DAG production. PA is also an important signaling molecule in the regulation of lipid metabolism. High levels of PA lead to the reduced translocation of the transcription regulator Opi1 to the nucleus [12], preventing its binding to the transcription factor Ino2. Because Ino2 is an activator of many fatty acid and phospholipid biosynthesis genes, an increase in PA directly upregulates fatty acid biosynthesis mechanisms [4,13]. Thus, modifying the pathway to increase the amount of PA in the cell does not affect PL production.

Subsequently, the pull and push strategy was tested. The strain PS05 was constructed by the overexpression of the *CDS* gene to pull C-flux into the PL biosynthesis pathway in combination with the overexpression of the phospholipid methyltransferase gene (*OPI3*) responsible for the final step of the phospholipid synthesis pathway, pushing the C-flux to the final product—phosphatidylcholine [14]. This strategy led to an increase in the total phospholipid content to 360% compared to the wild-type strain W29 when grown on glucose (W29—25 mg/g DCW, PS05—92.6 mg/g DCW), and to 190% when grown on glycerol as a carbon source (W29—21.45 mg/g DCW, PS05—47.5 mg/g DCW) [see Figure 2A–C]. Furthermore, the level of PC was improved almost four-and-a-half-fold to reach 47.3 mg/g on glucose and five-and-a-half-fold to reach 26.95 mg/g on glycerol.

In the next step, the strain PS05 was a subject of gene deletion, to remove genes responsible for partial phospholipid degradation. This step included genes encoding phospholipid diacylglycerol acyltransferase *LRO1* (YALI0E16797g) and phospholipase D *SPO14* (YALI0E18898g), creating the strains PS06 and PS07, respectively. The product of the *LRO1* gene converts DAG to TAG by transferring the acyl chain from the sn-2 position of glycerophospholipids [15]; hence, the deletion of this gene seemed quite important in increasing PL synthesis. In contrast, the role of *SPO14* has so far been well studied in the yeast *S. cerevisiae* where it is responsible for the continuous hydrolysis of phosphatidylcholine to choline and PA. However, the genes encoding phospholipases in *Y. lipolytica* have not been functionally characterized. Therefore, the aim of creating the strain PS07 was to investigate the effect of the *SPO14* deletion on phospholipid accumulation. The obtained results indicate that the PS06 strain with a *LRO1* deletion showed a decrease in phospholipid content by more than half compared to the PS05 strain using both glucose and glycerol (GLC—44.9 mg/g of PL, GLY—16.65 mg/g of PL). However, we also observed an increased number of TAGs on TLC plates [Figure S1A,B]. In turn, the strain PS07 showed the highest PL production when cultured on glucose. Compared to W29, the level of PC exhibited a nearly sixfold increase to 59.1 mg/g DCW. Furthermore, the total phospholipid content increased to 98.80 mg/g DCW, representing almost 390% in comparison to the strain W29 [Figure 2A]. When cultured on glycerol, the strain PS07 showed no significant improvement in total phospholipid content; only the level of PC increased almost threefold to 13.6 mg/g DCW compared to the W29 strain.

In summary, the performed genetic modifications allowed us to significantly improve the production of phospholipids by the corresponding transformants on both of the analyzed substrates, glucose and glycerol; however, the improvement on glucose was more pronounced. This means that in *Y. lipolytica,* the carbon source is an important aspect for phospholipids biosynthesis. Since the main objective of this study was to synthesize phospholipids from cheap and renewable substrate such as glycerol, two strains, PS05 and PS07, showing the highest phospholipid production on that substrate, were chosen for further experiments.

### 2.2. Enhancing Phospholipid Production by Overexpression of DGK1 and GUT1 Genes

To increase phospholipid production by the strains PS05 and PS07, we further modified these strains through the overexpression of DGA kinase *DGK1* (YALI0F19052g) and glycerol kinase *GUT1* (YALI0F00484g) genes, creating the strains PS08 and PS09, respectively. The product of the *DGK1* gene converts DAG, formed by TAG hydrolysis in the stationary phase, into PA, the major substrate of phospholipids [16]. Furthermore, this gene may also counteract the product of *PAH1*, causing the inhibition of TAG synthesis, thereby increasing the PA pool in the cell, as observed in *S. cerevisiae* [17]. In contrast, the overexpression of *GUT1* was carried out to increase the efficiency of glycerol assimilation by *Y. lipolytica*.

**Figure 3:**
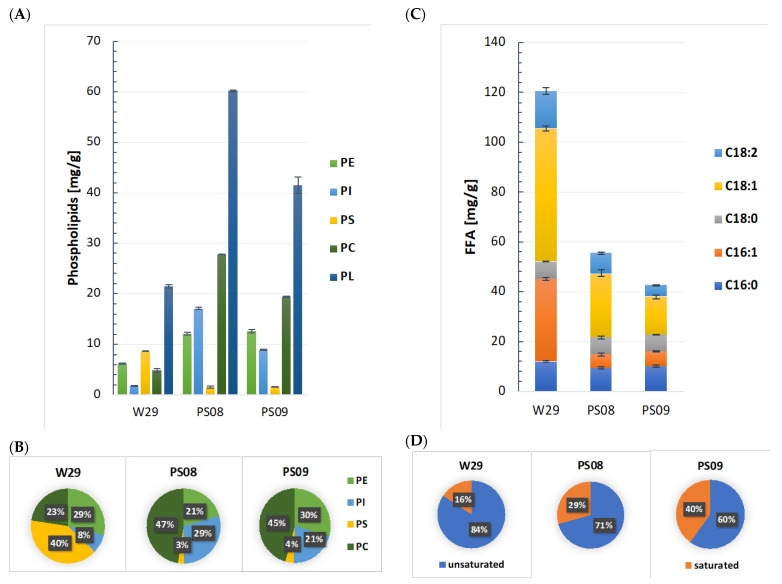
(**A**) Synthesis of phospholipids by final engineered *Y. lipolytica* strains. (**B**) Phospholipid classes distribution in strains W29, PS08, PS09. Cultivations were carried out for 144 h in shake flasks containing YNB with 60 g/L glycerol, C/N 99. (**C**) Total FA quantification of PS08 and PS09 compared with strain W29. (**D**) Distribution of saturated and unsaturated C16 and C18 FAs in the three strains.

The resulting strains were grown in YNB glycerol medium at C/N 99. Compared to W29, the level of PC showed a nearly sixfold increase, reaching 27.8 mg/g in the strain PS08. Furthermore, the PL content increased to 60.2 mg/g DCW, representing an almost 280% improvement compared to the control strain [Figure 3A]. A slightly lower production of PL was observed for the PS09 strain, which led to a 198% increase in PL produced (41.5 mg/g DCW) compared to the wild type. The level of PC was improved by fourfold to 19.4 mg/g DCW. The titer of PLs in both strains, PS08 and PS09, reached 409.36 mg/L and 290.5 mg/L, respectively. However, an almost twofold decrease of the dry biomass of the modified strains compared to the wild-type strain was observed [Figure S2]. In 2020, Ti Liu and co-workers developed a *Saccharomyces cerevisiae* platform for *de novo* production of oleoylethanolamide, a phospholipid derivative [18]. The research began with the construction of a strain overproducing phospholipids, which had a number of genetic modifications: an improved acyl-CoA supply resulting from the installation of a citrate shuttle, an enhanced fatty acid synthase system alongside the removal of acyl-CoA-degrading fatty acyl-CoA oxidase, the overexpression of the two main endogenous fatty acyl-CoA synthetases (*Faa1p* and *Faa4p*), and the deletion of the monoglyceride lipase-encoding gene *YJU3* and the *PAH1* gene. This strain produced 470 mg/L PL including 260 mg/L of PC. Similar modification would likely further improve the titers that can be achieved in *Y. lipolytica*, and they should be tested in the future.

The phospholipid composition changed due to the genetic modifications in the strains PS08 and PS09 compared to the control strain; PC content highly increased in both strains, up to 47% and 45%, and PS strongly decreased, to 3% and 4%, respectively [Figure 3B]. This was an expected effect caused by the overexpression of the *OPI* gene. The expected results were also confirmed by the decreased level of PE (21%) in the strain PS08. In contrast, in the PS09 strain, the PE level increased to 30% (compared to PS08), which may indicate a higher preference of *SPO14* toward PE hydrolysis. Unexpectedly, the proportion of PI in both strains increased (to 29% in PS08 and to 21% in PS09); however, the phenotype and consequences of this observation need further investigation.

Analysis of the total fatty acid (TFA) content in the cell showed more than a twofold decrease; PS08 and PS09 strains produced 55.5 mg/g and 42.5 mg/g of TFA, respectively, compared to the wild-type W29 strain, which produced 120.5 mg/g [Figure 3C]. This may indicate that, in *Y. lipolytica,* most of the fatty acids comes from the hydrolysis of TAGs, and in the case of the PS08 and PS09 strains, where TAG production is limited, a decrease in TFA content was noted. Furthermore, previous studies have shown that phospholipids in *Y. lipolytica* mostly incorporate saturated fatty acids at both the sn-1 and sn-2 positions [19]. This observation is consistent with the results shown in Figure 3D, where the percentage of saturated FAs between W29 and PS08, or PS09, increased from 16% to 29% and 40%, respectively.

### 2.3. Optimization of Flask Culture Conditions

**Figure 4:**
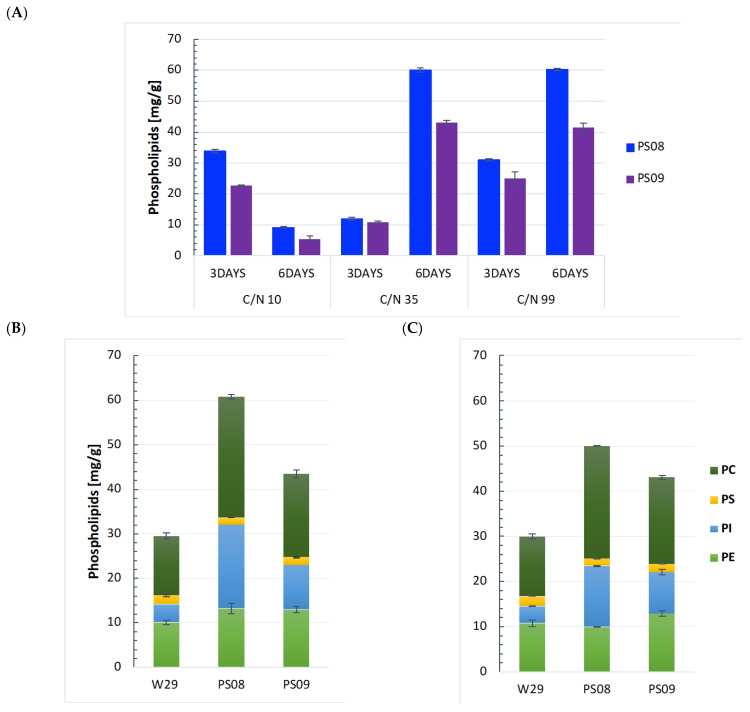
Effects of different culture conditions on *Y. lipolytica*. (**A**) Effects of different time of cultivation (72 h/144 h) and C/N ratios on PLs production. (**B**,**C**) Production of PLs after 144 h with the twofold addition of glucose (**B**) and gluconate (**C**). Supplementation of these two substrates accounted for approximately 5% of the total carbon consumed by the cells and was added at the beginning and middle of the culture; the primary carbon source was glycerol. Error bars represent standard deviation from at least three biological replicates.

For the lipid accumulation process in yeast, nitrogen limitation usually determines the biomass content, whereas the carbon source concentration usually determines the lipid amount [20]. Therefore, the C/N ratio is important in determining the lipid content and biomass number of oleaginous microorganisms. According to many studies conducted in this field, the C/N ratio not only affects lipid accumulation but is also a strain-dependent factor for the *Y. lipolytica* [21]. Related to strain differences, the choice of a good carbon source is also of high importance [22]. Therefore, to achieve high lipid accumulation, the initial C/N molar ratio for PL biosynthesis was optimized. We tested three different C/N ratios—10, 35, and 99—using glycerol as a carbon source. Moreover, since phospholipids are a class of lipids that enter the membrane structures of cells, and thus their synthesis is required during each phase of cell growth, we analyzed their synthesis at different time-points during the cultivation (after 3 and 6 days of the process). Initially, our hypothesis assumed, that in the late stationary phase, when the cell begins to accumulate lipids, the main lipid substrates are directed at the synthesis of FAs and TAGs—not PL. As shown in [Figure 4A], nearly the same PL yield in both modified strains was obtained with a C/N ratio of 35 and 99 at day 6, reaching about 60 mg/g in the strain PS08 and about 41 mg/g in the strain PS09. At C/N 99, PL production starts earlier than at C/N 35, which is evident after day 3. These results indicate that the optimal C/N molar ratio is in the range between 35 and 99 promoted phospholipid accumulation when *Y. lipolytica,* with the modified phospholipid pathway, was cultured on glycerol.

Furthermore, as describe above [Figure 2A,B], the synthesis of PL from glycerol, despite glycerol being the phospholipid backbone, is less efficient than from glucose. This led us to speculate that genes involved in the phospholipid biosynthetic pathway may be partially dependent on NADPH, whose generation in *Y. lipolytica* is mainly through the oxidative pentose phosphate pathway (PPP), which is somewhat attenuated when utilizing only glycerol [23]. Thus, our second strategy to optimize the medium for PL production was to culture *Y. lipolytica* on glycerol and feed limiting quantities of glucose or gluconate as ‘dopant’ substrates to augment the reductive metabolism of lipogenesis, through obligate NADPH synthesis. This method worked well in a study by Park et al. (2019), who used gluconate dosing in *Y. lipolytica* to accelerate acetate-driven lipogenesis and glucose dosing in *Moorella thermoacetica* to stimulate the reduction of CO_2_ to acetate by increasing pyruvate kinase-mediated ATP synthesis [24]. Our results show that neither glucose nor gluconate addition affected PL synthesis by the modified strains [Figure 3A and Figure 4B,C]. All of the above-described results raised many questions about the PL biosynthesis by *Y. lipolytica* using different carbon sources, which need further studies for their elucidation.

### 2.4. Production of Phospholipids on Bioreactor Scale

**Figure 5:**
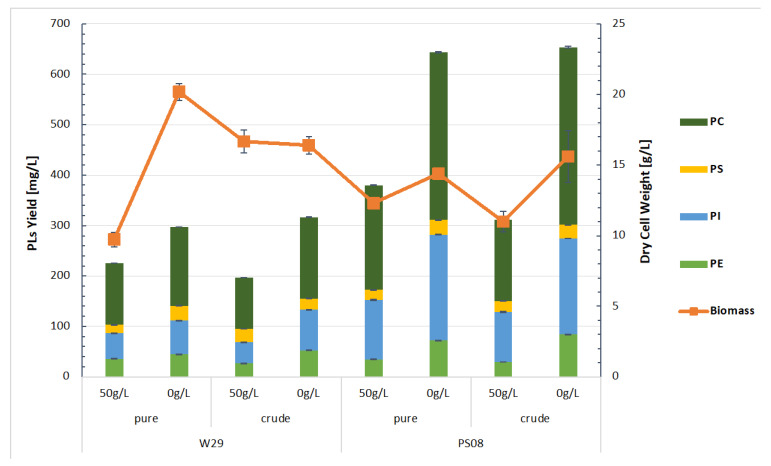
Phospholipids production by control strain and PS08 using pure/crude glycerol as a substrate in bioreactor. Cultures containing YNB medium with 100 g/L glycerol were carried out until complete substrate consumption.

The biotechnological approach in designing an efficient process needs to be evaluated on a larger scale. Thus, further experiments were performed using the PS08 strain showing the highest PL synthesis and the wild-type strain W29 as a control, which were cultured in a bioreactor using pure and waste glycerol as a carbon sources. Biomass and phospholipid content were determined at the moment of partial (50 g/L) and complete (0 g/L) substrate consumption. The obtained results are shown in Figure 5.

PL production was at a similar level regardless of the type of glycerol used as a carbon source (pure or crude). This observation was made for both W29 and PS08 strains. It demonstrates that there is no negative effect of trace amounts of impurities found in crude glycerol on phospholipid synthesis. After the completion of the bioreactor culture, the PS08 strain’s PL content was almost 160% higher than the PL content observed during growth in flasks. At the same time, the PC content increased by nearly 180% (PL—643.7 mg/L, PC—332.6 mg/L from pure glycerol, PL—653.7 mg/L, PC—352.6 mg/L from crude glycerol) [Figure S3]. In the case of the wild-type strain, there was no increase in the production of PL compared to the flask cultures (PL—296 mg/L, PC—155 mg/L from pure glycerol, PL –316 mg/L, PC—160,7 mg/L from crude glycerol) [Figure S3]. Moreover, this strain maintained phospholipid synthesis at a fairly constant level regardless of the growth phase, assuming that 50 g/L glycerol corresponds to the early stationary phase and total glycerol consumption corresponds to the late stationary phase of growth. In contrast, for the strain PS08, one can see an approximately twofold increase in PL content at the time of total substrate consumption, indicating that intensive phospholipid accumulation occurs during the late stationary phase along with the typical accumulation of other lipid fractions characteristic of oleaginous microorganisms [8].

These results indicate that *Y. lipolytica*, with just the overexpression of the minimal set of enzymes, was already able to produce almost 650 mg/L of phospholipids, using technical glycerol, as well as waste glycerol, as a carbon source.

## 3. Materials and Methods

### 3.1. Strains, Media, and Culture Conditions

The strains used in this study are shown in Table S1 (Supplementary Materials). The strains of *Y. lipolytica* were maintained in a YPD medium consisting of 10 g/L yeast extract, 10 g/L peptone, and 20 g/L glucose with 10 g/L agar (for plates) at 28 °C. Minimal (YNB) medium for the selection of the *Y. lipolytica* transformants was prepared using 1.7 g/L yeast nitrogen base (without amino acid and ammonium sulfate, Sigma-Aldrich, Saint Louis, MI), 20 g/L glucose, 5 g/L NH_4_Cl, and 50 mM phosphate buffer pH 6.8 with 20 g/L agar. The *Escherichia coli* strains harboring plasmids were cultured overnight in LB medium (5 g/L yeast extract, 10 g/L tryptone, and 10 g/L NaCl with 20 g/L agar in plates and 0.05 mg/L kanamycin) at 37 °C. For long-term storage, the strains were kept at −80 °C in 500 g/L glycerol.

### 3.2. Plasmid Preparation

In this study, two types of plasmids were used. Gene disruption-carrying cassettes were constructed using the pCR-Blunt II TOPO vector (Invitrogen, Carlsbad, CA, USA), whereas plasmids used for gene overexpression were based on a JMP62 plasmid [25]. The disruption cassettes were prepared as described by Fickers and co-workers [26]. Briefly, 1 kb fragments representing promoter (P) and terminator (T) sequences of *SPO14* (*YALI0E18898g*) and *LRO1* (*YALI0E16797g*) were amplified by PCR from *Y. lipolytica* W29 genomic DNA and subsequently fused using the PCR-fusion technique. The obtained PT fragments were cloned into the pCR-Blunt II TOPO vector. Resulting plasmids were then digested with the I-*Sce*I restriction enzyme, and the I-*Sce*I digested *URA3ex* (Ura) or *LEU2ex* (Leu) marker was inserted to obtain the disruption cassettes. The overexpression cassettes were constructed by the PCR amplification of selected genes involved in the PL synthesis pathway from the genomic DNA of *Y. lipolytica* W29. PCR fragments obtained with the appropriate restriction enzyme sites were then digested and ligated into the *Bam*HI/*Bgl*II and *Xma*JI (*Avr*II) digested JMP62 vector carrying a strong constitutive TEF promoter. All constructs were verified using PCR and DNA sequencing (Genomed S.A, Warsaw, Poland). Plasmids and primers used in this study are listed in Tables S2 and S3 (Supplementary Materials).

### 3.3. Cloning and Transformation Protocols

All restriction enzymes, the Phusion high-fidelity DNA polymerase, and the T4 DNA ligase were purchased from ThermoScientific (Waltham, MA, USA). The reactions followed standard protocols as described by the manufacturers. Plasmids from *E. coli* were extracted using the Plasmid Mini Kit (A&A Biotechnology, Gdańsk, Poland). DNA purification from the gel was carried out with the Gel Out extraction kit (A&A Biotechnology, Gdańsk, Poland).

Competent *E. coli* DH5α cells were transformed by the thermal shock protocol. Yeast transformation was carried out following the lithium-acetate method [27]. Transformants were selected on YNB-Leu, YNB-Ura, or YNB-Hygro media, depending on their genotype.

### 3.4. Flask Cultivation

Three transformants of each strain were tested. The preculture was conducted in 50 mL of rich YPD medium at 28 °C with 250 rpm of agitation for 48h. After that time, cells were washed twice with sterile distilled water and used for inoculation. The initial OD_600_ was set at 0.5 for each strain. Shake flask cultivations were performed in minimal medium containing 60 g/L glucose or glycerol, 1.3 g/L (NH_4_)_2_SO_4_, 1 g/L MgSO_4_x7H_2_O, 1.7 g/L YNB, and 50 mM phosphate buffer pH 6.8 and cultivated at 160 rpm at 28 °C for 144 h. Experiments were conducted in three biological replicates.

### 3.5. Bioreactor Cultures

The precultures were prepared as described for flask cultures. The initial OD_600_ was set to 0.5. The batch cultures were conducted in lipid synthesis medium, as described above, containing 100 g/L of pure or crude glycerol. Crude glycerol was derived from biofuel production from the Trzebinia refinery (LOTOS Group) with 86% (*v*/*w*) glycerol content. All cultures were performed in a 5 L stirred-tank reactor (BIOSTATB-PLUS, Sartorius, Germany) with a working volume of 2 L at 28 °C. The aeration was set to 0.8 vvm with a stirring rate of 600 rpm. The pH 6.8 was maintained automatically by the additions of 30% (*w*/*v*) NaOH solution. The cultures were terminated when the carbon source was completely consumed. The experiments were performed in biological duplicates.

### 3.6. Lipids Extraction

The lipids were extracted from lyophilized biomass using a modified method, as described before [28]. Briefly, 100 mg of biomass was weighed and mixed with a 2:1 *v*/*v* chloroform-methanol solution (5 mL) and left overnight at 80 °C with 600 rpm of rotation. Then, MgCl_2_ was added to the suspension at a concentration of 0.034% and vortexed for 10 min. Samples were centrifuged at 1000× *g* for 3 min. The aqueous upper phase was discarded. The artificial upper phase, methanol-water-chloroform (48:47:3), was added to the samples and mixed. Samples were centrifuged at 1000× *g* for 3 min. The aqueous upper phase was again discarded, and the organic lower phase containing the lipid fraction was transferred to a new tube. The solvent was evaporated, and the collected lipids were stored at −20 °C for further analysis.

### 3.7. Quantification of Phospholipids

The quantitative content analysis of individual phospholipid fractions (phosphatidylethanolamine, phosphatidylinositol, phosphatidylserine, and phosphatidylcholine) in the obtained extracts was determined by high-performance liquid chromatography using DIONEX UltiMate 3000 chromatograph from Thermo Scientific (Waltham, MA, USA) equipped with a UV/CAD detector (at 300 nm) and a BetaSil DIOL column (Thermo Scientific, 150 × 4.6 mm, 5 µm). In this analysis, the injection volume was 10 µL, whereas the autosampler and column temperature were 20 °C and 30 °C, respectively. The elution was set at a constant flow (3 mL/min) and performed in a gradient: solvent A (1% HCOOH, 0.1% triethylamine (TEA) in water), solvent B (hexane), and solvent C (2-propanol). The elution program was as follows: 3/40/57 (%A/%B/%C (*v*/*v*/*v*)), in 5 min = 4/40/56, in 6 min = 5/40/55, in 7 min = 6/40/54, in 8 min = 7/40/53, and then constant for up to 18 min. The total analysis and conditioning time was 20 min.

### 3.8. Quantification of Fatty Acids

Samples for total fatty acid analysis were taken as 5 mL of culture at the end of shake flask or bioreactor cultivations. The 5-mL culture volume was kept at −80 °C and then freeze dried. The total lyophilized culture was processed for fatty acid extraction and derivatization to methyl esters (FAMEs), using the method described before [29]. In short, 10 mg of biomass was mixed with 2 mL of solvent solution: 2.5% H_2_SO_4_, 97.5% methanol, 50 μg/mL of C17:0 as an internal standard, in Pyrex glass tubes (Sigma-Aldrich, Saint Louis, MI, USA). Then all samples were thoroughly mixed and incubated at 80 °C overnight to form FAMEs. FAMEs were extracted by hexane and 0.9% NaCl. The organic phase was collected and stored at −20 °C until use. FAME analysis was performed by gas chromatography on a GC-MS instrument (Shimadzu, Kyoto, Japan) equipped with a Zebron ZB-FAME capillary column (30 m × 0.25 mm × 0.20 µm). The samples (1 µL at 250 °C) were injected in splitless mode using helium (1 mL min^−1^). The identification of fatty acids was carried out by the comparison of retention times with reference compounds (Supelco 37 Component FAME Mix, Sigma-Aldrich).

### 3.9. Thin-Layer Chromatography

The qualitative estimation of phospholipids and neutral lipids extracted from *Y. lipolytica* was carried out by the thin layer chromatography method [30] with a solvent system of chloroform-methanol-water (32.5:12.5:2) and petroleum ether/diethylether/acetic acid (32:8:0.8), respectively.

## 4. Conclusions

In this study, we investigated the potential of *Y. lipolytica* to produce phospholipids, especially phosphatidylcholine. Interestingly, we found that increasing the amount of phosphatidic acid by manipulating the upstream lipid pathway and presumably targeting it in the phospholipid pathway does not lead to an increase in their synthesis. The very wide optimal C/N ratio ranging from 35 to 99 for phospholipid production is also interesting. Finally, the best strain (PS08), containing only a few overexpressed genes, was able to produce, under the best fermentation conditions, almost 650 mg/L PL, including 335 mg/L PC using waste glycerol, as well as technical glycerol, as the sole carbon source. This study shows that relatively simple pathway engineering in *Y. lipolytica* can lead to an increased number of phospholipids, which can most likely be further improved by manipulating the remaining parts of the lipid pathways, the gene copy numbers, and precursor availability. These results also suggest that *Y. lipolytica* may be a promising host for the production of other interesting phospholipid-derived compounds.

## Supplementary Materials

The following supporting information can be downloaded at: www.mdpi.com/article/10.3390/ijms231810737/s1. References [31,32] are cited in the supplementary materials.
