# Supplementary material for: Elevating Phospholipids Production Yarrowia lipolytica from Crude Glycerol
Source: Int J Mol Sci. 2022 Sep 14;23(18):10737. doi: 10.3390/ijms231810737 (PMC9505966; doi:10.3390/ijms231810737)
Supplement: Supplementary file 1 [file ijms-23-10737-s001.zip › ijms-1883686-supplementary.pdf]

SUPPLEMENATRY MATERIALS

FIGURES

A)

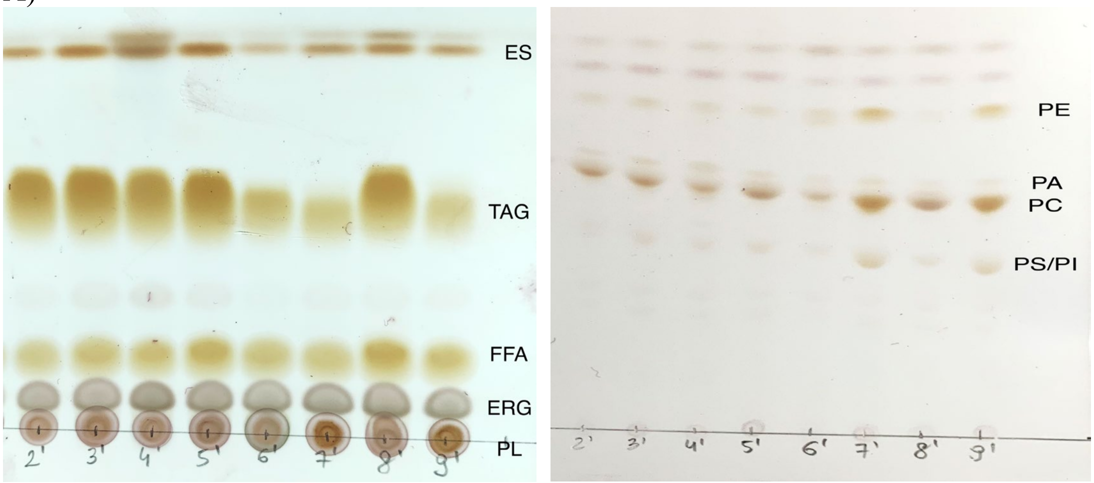

B)

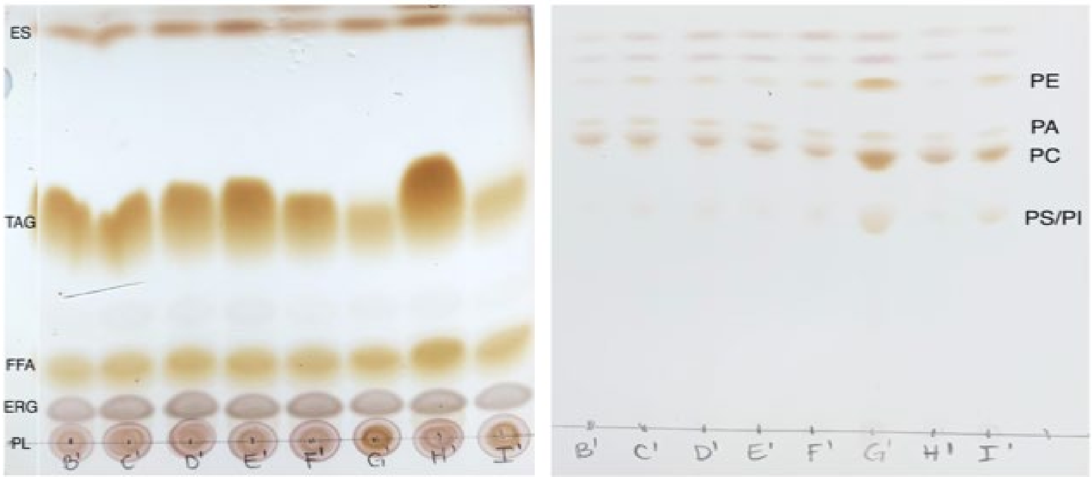

Figure S1. A) TLC separation of neutral lipids (left side) and phospholipids (right side) from *Y. lipolytica* strains grown on glucose. 2'-W29, 3'-PS01, 4'-PS02, 5'-PS03, 6'-PS04, 7'-PS05, 8'-PS06, 9'-PS07 B) TLC separation of neutral lipids (left side) and phospholipids (right side) from strains grown on glycerol. B'-W29, C'-PS01, D'-PS02, E'-PS03, F'-PS04, G'-PS05, H'-PS06, I'-PS07. FFA, free fatty acid; ERG, ergosterol; ES, sterol esters.

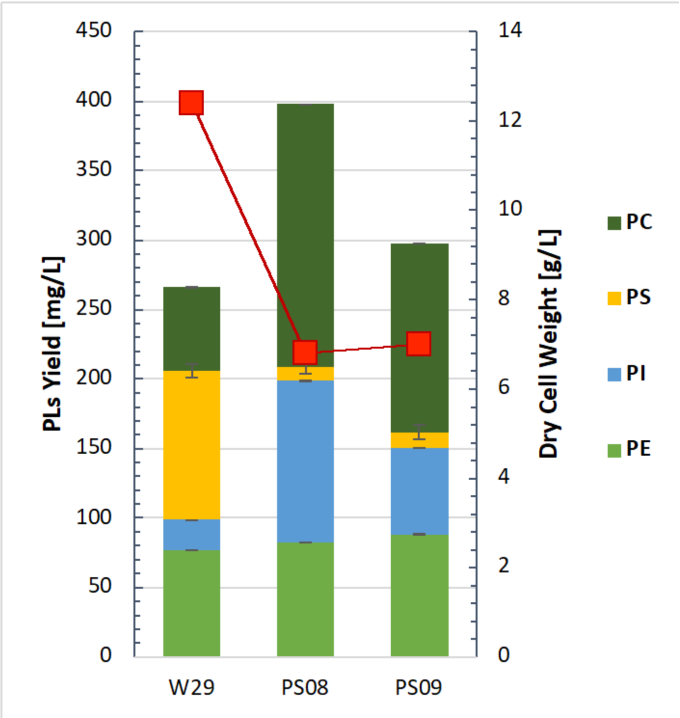

Figure S2. Titers of phospholipids in final engineered *Y. lipolytica* strains. Cultivations were carried out for 144 h in shake flasks containing YNB with 60 g/L glycerol, C/N 99.

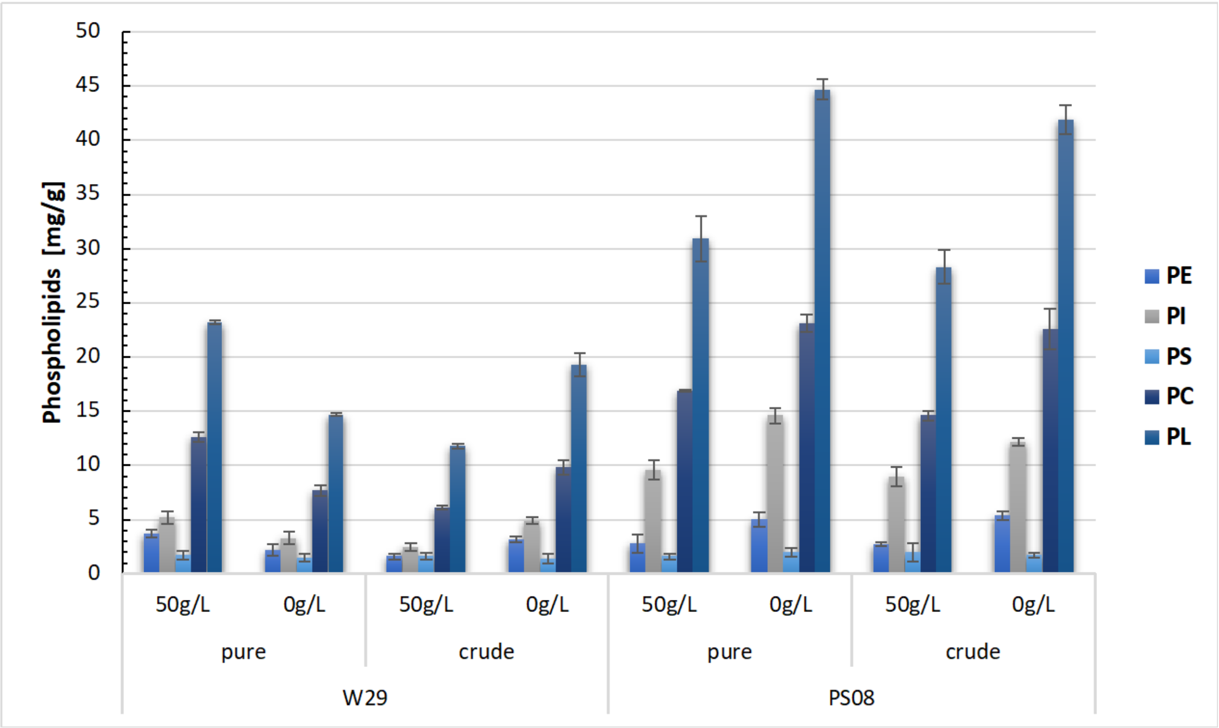

Figure S3. Phospholipids production by control strain and PS08 using pure/crude glycerol as a substrate in bioreactor. Cultures containing YNB medium with 100 g/L glycerol, were carried out until complete substrate consumption.

TABLES

Table S1. Strains of *Y. lipolytica* used in this study.

Strain	Genotype	Source or reference
W29	*MATa WT*	Culture collection of the Department of Biotechnology and Food Microbiology
Po1d	*MATa ura3-302 leu2-270 xpr2-322*	CLIB139
PS01	*MATa ura3-302 leu2-270 xpr2-322* pTEF-*CDS*	This study
PS02	*MATa ura3-302 leu2-270 xpr2-322* pTEF-*CDS* pTEF-*SCT*	This study
PS03	*MATa ura3-302 leu2-270 xpr2-322* pTEF-*CDS* pTEF-*SLC*	This study
PS04	*MATa ura3-302 leu2-270 xpr2-322* pTEF-*CDS* pTEF-*ALE*	This study
PS05	*MATa ura3-302 leu2-270 xpr2-322* pTEF-*CDS* pTEF-*OPI*	This study
PS06	*MATa ura3-302 leu2-270 xpr2-322* pTEF-*CDS* pTEF-*OPI △LRO1*	This study
PS07	*MATa ura3-302 leu2-270 xpr2-322* pTEF-*CDS* pTEF-*OPI △SPO14*	This study
PS08	*MATa ura3-302 leu2-270 xpr2-322* pTEF-*CDS* pTEF-*OPI* pTEF-*DGK*, pTEF-*GUT1*	This study
PS09	*MATa ura3-302 leu2-270 xpr2-322* pTEF-*CDS* pTEF-*OPI* pTEF-*DGK*, pTEF-*GUT1 △SPO14*	This study

Table S2. Plasmids used in this study.

Plasmid	Description	Reference
JME1046	JMP62-URA3ex, TEFp, KanR	[31]
JME1046	JMP62-LEU2ex, TEFp, KanR	[32]
JMP62-CDS	JMP62-URA3ex-pTEF-*CDS*	This study
JMP62-ALE	JMP62-URA3ex-pTEF-*ALE*	This study
JMP62-SCT	JMP62-URA3ex-pTEF-*SCT*	This study
JMP62-SLC	JMP62-LEU2ex-pTEF-*SLC*	This study
JMP62-OPI3	JMP62-LEU2ex-pTEF-*OPI3*	This study
JMP62-DGK	JMP62-LEU2ex-pTEF-*DGK1*	This study
JMP62-GUT	JMP62-*Hygro*ex-pTEF-*GUT1*	This study
PUT-LRO	pCR™-Blunt II TOPO™ PUT-*LRO1*	This study
PLT-SPO14	pCR™-Blunt II TOPO™ PLT-*SPO14*	This study

Table S3. Primers used in this study.

**Primer**	**Sequence (5' -> 3')**	**Aim**
P1-LRO1-F	ATTAGCGGCCGCACCAGACTTGCTCCACATTC	Knock-out of the LRO1 and SPO14 genes
P2-LRO1-R	CGATTACCCTGTTATCCCTACCACCGGAAAAAAGCCGATTAC	
T1-LRO1-F	GGTAGGGATAACAGGGTAATCGGATGGAGAAGGGCGTTCG	
T2-LRO1-R	ATTAGCGGCCGCCACGGCTTGCTTTCAGATTC	
P1-SPO14-F	ATTAGCGGCCGCAGCTTTCCTGCAGCATGAG	
P2-SPO14-R	CGATTACCCTGTTATCCCTACCTTTGTGGCGACTCAAGAGTG	
T1-SPO14-F	GGTAGGGATAACAGGGTAATCGGCGACAAACACGTGCTAAG	
T2-SPO14-R	ATTAGCGGCCGCAAACTTGTTCGACGCCATTC	
LRO1-ver-F	GAGTCGCACTTCAGAAAGC	Verification of the knock-out
LRO1-ver-R	CATGGAGCCGGAAATGTC	
SPO14-ver-F	CTCCACTCCCCAGATGAAC	
SPO14-ver-R	CGAGTTGAAGGGGTTCTTG	
OPI3-BamHI-F	GAGAGGATCCATGTCCTTTCCTGACAAGATTGTTG	Overexpression cassetes
OPI3-AvrII-R	TCTCCCTAGGTTACTTCTGCTTGGCAGCGAG	
DGK1-BamHI-F	GAGAGGATCCATGTCTGCTGCATCTACTGGAG	
DGK1-AvrII-R	TCTCCCTAGGTTACTTCTTGAAGATGTCCAGCAG	
CDS1-BamHI-F	GAGAGGATCCATGTCTGAAAAAATTGACGCCCACAC	
CDS1-AvrII-R	TCTCCCTAGGTTATTCAAGAACGGCGCAGAAGC	
ALE1-BamHI-F	GAGAGGATCCATGGCCTTTCCATGGGCAGATAAG	
ALE1-AvrII-R	TCTCCCTAGGTTACTTGGTCTTGATGGTGTCCTTCTTC	
SCT1-BamHI-F	GAGAGGATCCATGTCCGAAACCGACCATC	
SCT1-AvrII-R	TCTCCCTAGGTTATTCCTCATCCTGCTCTCGTC	
SLC1-BamHI-F	GAGAGGATCCATGTCCGTTGCATCCAAGCTC	
SLC1-AvrII-R	TCTCCCTAGGCTACTGAGTCTTCTGGCCAGCGTAG	
GUT1-BamHI-F	GAGAGGATCCATGTCTTCCTACGTAGGAGC	
GUT1-AvrII-R	TCTCCCTAGGTTACTCAAGCCAGCCAAC	
JMP62-pTEF-START	GGGTATAAAAGACCACCGTCC	Verification of overexpression cassetes integration
JMP62-61STOP	GTAGATAGTTGAGGTAGAAGTTG	
